# Supplementary material for: Enhanced protective efficacy of a thermostable RBD-S2 vaccine formulation against SARS-CoV-2 and its variants
Source: NPJ Vaccines. 2023 Oct 25;8:161. doi: 10.1038/s41541-023-00755-2 (PMC10600342; doi:10.1038/s41541-023-00755-2)
Supplement: Supplementary file 1 — Supplementary Information [file 41541_2023_755_MOESM1_ESM.pdf]

Supplementary Information

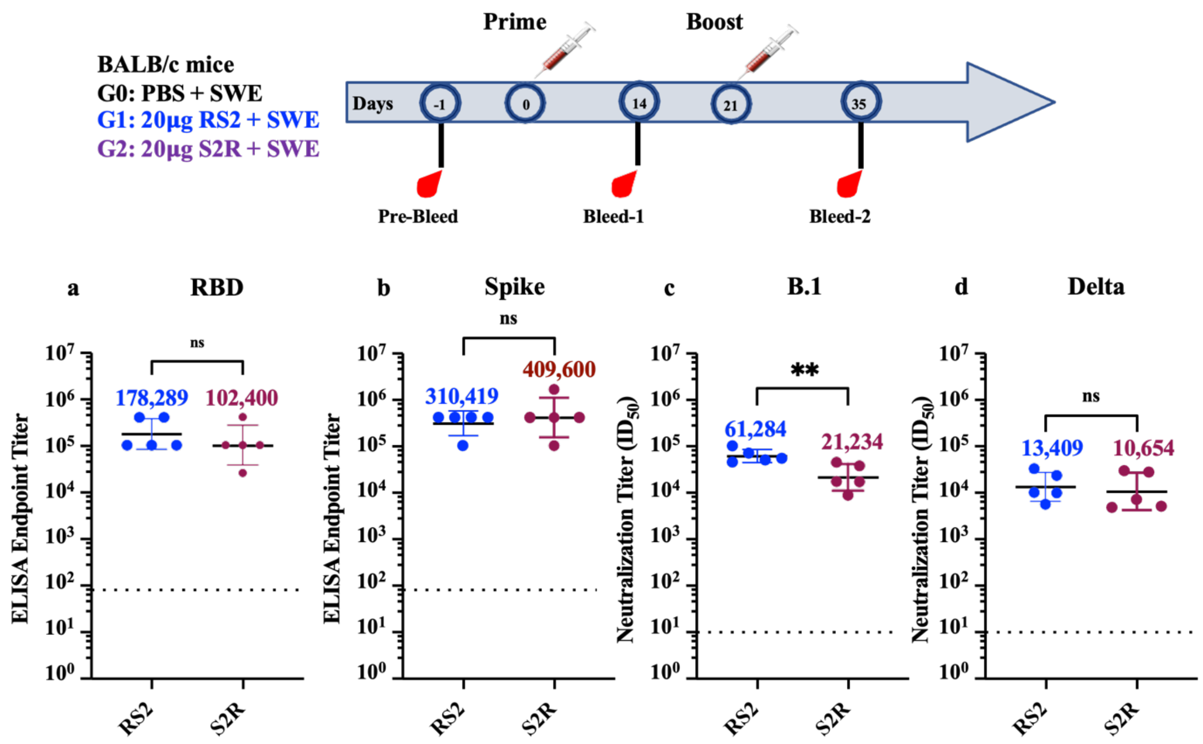

Supplementary Figure 1. Comparative immunogenicity of RS2 and S2R in mice. BALB/c mice were immunized twice with 20µg of RS2 or S2R. Two weeks following boost vaccination RBD and Spike specific IgG levels and neutralizing antibody titers were measured in immunized mice sera samples. Comparative ELISA endpoint titers against **(a)** RBD and **(b)** Spike ectodomain, respectively **(c and d)** Comparative neutralizing antibody titers elicited by 20µg of RS2 and S2R against **(c)** B.1, **(d)** Delta variant pseudoviruses. Titers are shown as geometric mean with geometric SD. The ELISA binding and neutralization titers were analyzed with a two-tailed Mann–Whitney test. (ns indicates non-significant, * indicates $p < 0.05$, ** indicates $p < 0.01$, **** indicates $p < 0.0001$).

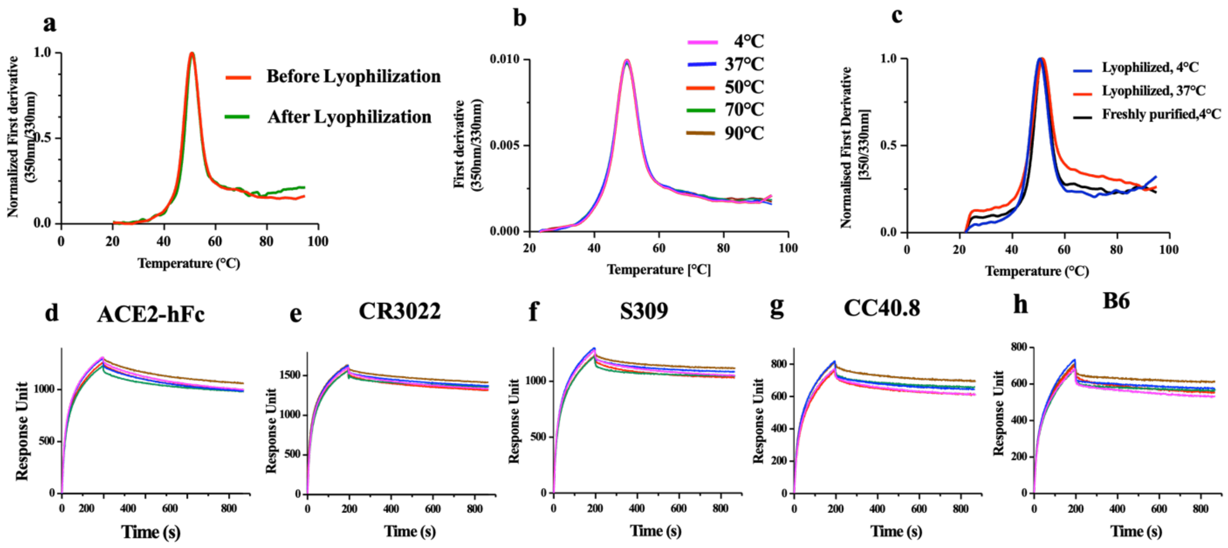

Supplementary Figure 2. Effect of transient thermal stress on lyophilized RS2. RS2 protein was dialyzed in water and then lyophilized. **(a)** Equilibrium thermal unfolding profile of RS2 before and after lyophilization and resolubilization in PBS. Lyophilized RS2 was incubated for one hour at different temperatures (4, 37, 50, 70 and 90°C). Following reconstitution in PBS buffer, thermal stability of **(b)** RS2 was monitored using Nano-DSF. **(c)** Thermal stability of lyophilized and resolubilized RS2 after incubation at 37 °C for 1 month. **(d-h)** In addition, RS2 samples were characterized for binding of with panel of RBD **(d-f)** and S2 **(g and h)** specific antibodies using SPR **(d)** ACE2-hFc, **(e)** CR3022, **(f)** S309, **(g)** CC40.8, and **(h)** B6.

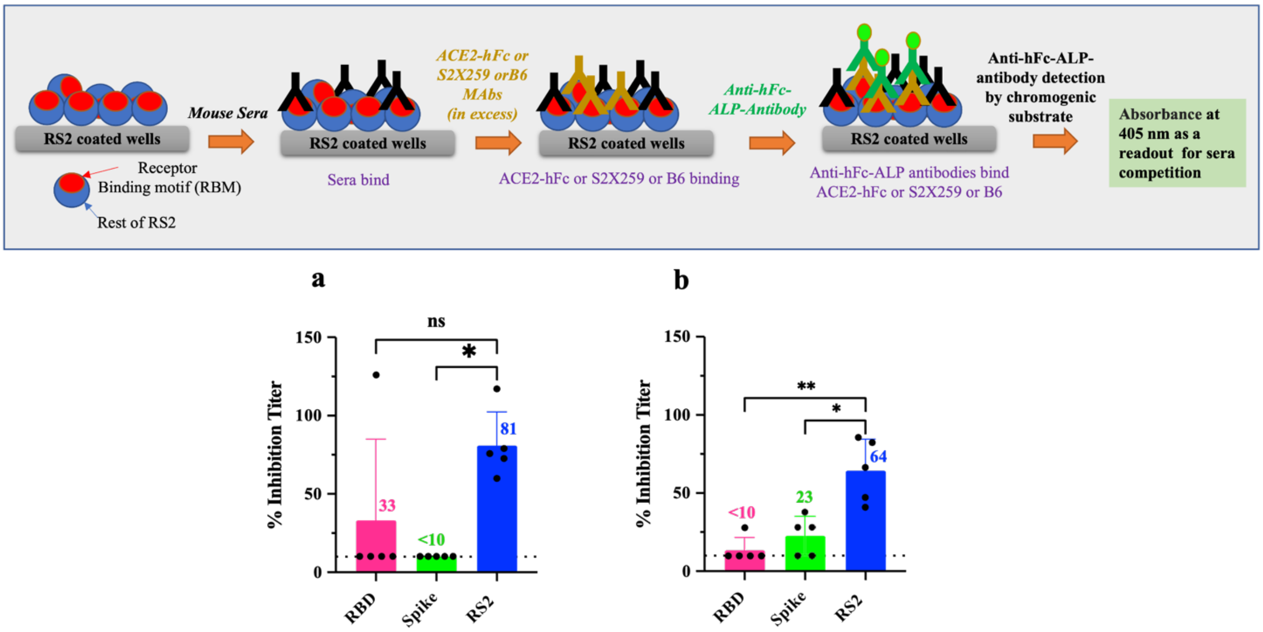

Supplementary Figure 3. Competition of immunized mice sera with RBD and S2 specific monoclonal antibodies. BALB/c mice were immunized with 20µg of RBD, Spike, or RS2. Top panel: Schematic of assay. Bottom panel **(a and b)** ELISA competition titers against **(a)** S2X259 (Class 4) and **(b)** B6 (S2 helix) monoclonal antibodies for individual, immunized mice sera. Immunogens used to elicit respective sera are listed on the x-axis and competition titers on the y-axis of each bar graph.

Source Data Figures

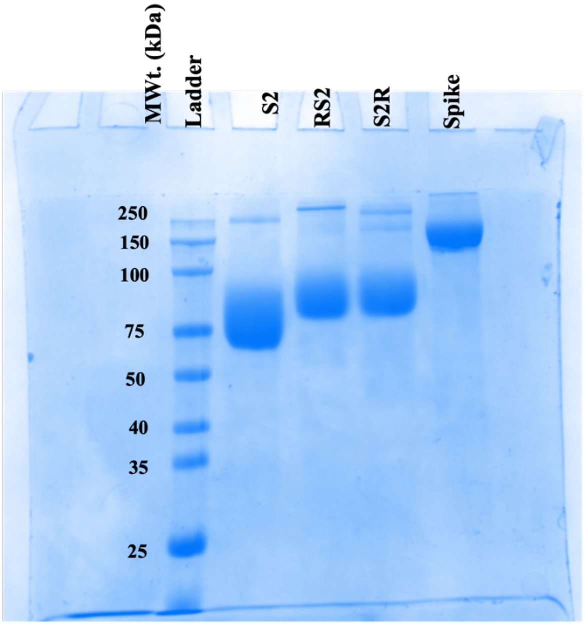

Uncropped gel image for Fig. 1b

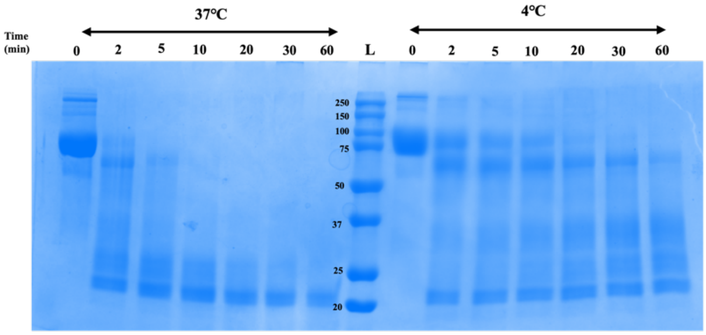

Uncropped gel image for Fig. 2i

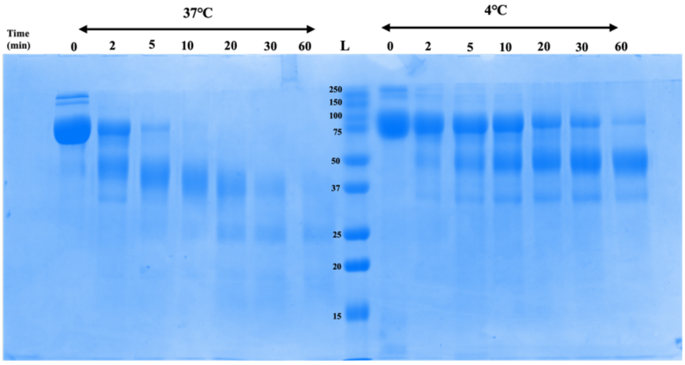

Uncropped gel image for Fig. 2j

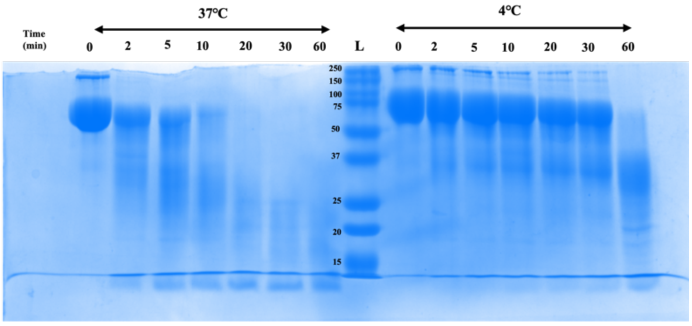

Uncropped gel image for Fig. 2k

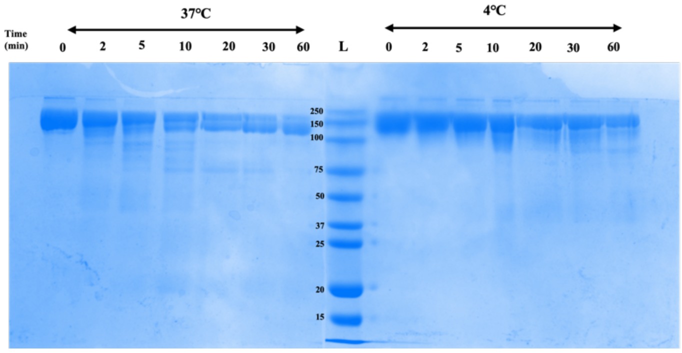

Uncropped gel image for Fig. 2l
