# Supplementary material for: Enhanced protective efficacy of a thermostable RBD-S2 vaccine formulation against SARS-CoV-2 and its variants
Source: NPJ Vaccines. 2023 Oct 25;8:161. doi: 10.1038/s41541-023-00755-2 (PMC10600342; doi:10.1038/s41541-023-00755-2)
Supplement: Supplementary file 2 — REPORTING SUMMARY [file 41541_2023_755_MOESM2_ESM.pdf]

nature portfolio

Corresponding author(s): Raghavan Varadarajan

Last updated by author(s): Aug 7, 2023

Reporting Summary

Nature Portfolio wishes to improve the reproducibility of the work that we publish. This form provides structure for consistency and transparency in reporting. For further information on Nature Portfolio policies, see our Editorial Policies and the Editorial Policy Checklist.

Statistics

For all statistical analyses, confirm that the following items are present in the figure legend, table legend, main text, or Methods section.

n/a	Confirmed	
☐	☒	The exact sample size (*n*) for each experimental group/condition, given as a discrete number and unit of measurement
☐	☒	A statement on whether measurements were taken from distinct samples or whether the same sample was measured repeatedly
☐	☒	The statistical test(s) used AND whether they are one- or two-sided<br>*Only common tests should be described solely by name; describe more complex techniques in the Methods section.*
☒	☐	A description of all covariates tested
☐	☒	A description of any assumptions or corrections, such as tests of normality and adjustment for multiple comparisons
☐	☒	A full description of the statistical parameters including central tendency (e.g. means) or other basic estimates (e.g. regression coefficient) AND variation (e.g. standard deviation) or associated estimates of uncertainty (e.g. confidence intervals)
☐	☒	For null hypothesis testing, the test statistic (e.g. *F*, *t*, *r*) with confidence intervals, effect sizes, degrees of freedom and *P* value noted<br>*Give P values as exact values whenever suitable.*
☒	☐	For Bayesian analysis, information on the choice of priors and Markov chain Monte Carlo settings
☒	☐	For hierarchical and complex designs, identification of the appropriate level for tests and full reporting of outcomes
☒	☐	Estimates of effect sizes (e.g. Cohen's *d*, Pearson's *r*), indicating how they were calculated

Our web collection on statistics for biologists contains articles on many of the points above.

Software and code

Policy information about availability of computer code

Data collection	No proprietary software was used
Data analysis	Graph Pad Prism 10.0

For manuscripts utilizing custom algorithms or software that are central to the research but not yet described in published literature, software must be made available to editors and reviewers. We strongly encourage code deposition in a community repository (e.g. GitHub). See the Nature Portfolio guidelines for submitting code & software for further information.

Data

Policy information about availability of data

All manuscripts must include a data availability statement. This statement should provide the following information, where applicable:

- Accession codes, unique identifiers, or web links for publicly available datasets
- A description of any restrictions on data availability
- For clinical datasets or third party data, please ensure that the statement adheres to our policy

All data are available in the main text or the supplementary materials.

Research involving human participants, their data, or biological material

Policy information about studies with human participants or human data. See also policy information about sex, gender (identity/presentation), and sexual orientation and race, ethnicity and racism.

Reporting on sex and gender	Not applicable (No human participants, their data, or biological material were involved in this study)
Reporting on race, ethnicity, or other socially relevant groupings	Not applicable (No human participants, their data, or biological material were involved in this study)
Population characteristics	Not applicable (No human participants, their data, or biological material were involved in this study)
Recruitment	Not applicable (No human participants, their data, or biological material were involved in this study)
Ethics oversight	Not applicable (No human participants, their data, or biological material were involved in this study)

Note that full information on the approval of the study protocol must also be provided in the manuscript.

Field-specific reporting

Please select the one below that is the best fit for your research. If you are not sure, read the appropriate sections before making your selection.

☒ Life sciences ☐ Behavioural & social sciences ☐ Ecological, evolutionary & environmental sciences

For a reference copy of the document with all sections, see nature.com/documents/nr-reporting-summary-flat.pdf

Life sciences study design

All studies must disclose on these points even when the disclosure is negative.

Sample size	Sample size for the animal studies was based on prior experience with similar animal studies carried out previously, see for example PMID 34956193 as well as other similar studies reported in the literature which typically use a group size of 5 animals.
Data exclusions	No data were excluded
Replication	With the exception of the animal immunogenicity and challenge studies, most measurements were made in duplicates or triplicates. For the animal studies, for reasons of cost and ethical approvals it was not possible to carry out replicates of entire studies.
Randomization	Animals were randomly allocated into different groups.
Blinding	In immunization and challenge studies, the animal handlers as well as individuals doing neutralization assays were blinded to the identity of the formulations used.

Reporting for specific materials, systems and methods

We require information from authors about some types of materials, experimental systems and methods used in many studies. Here, indicate whether each material, system or method listed is relevant to your study. If you are not sure if a list item applies to your research, read the appropriate section before selecting a response.

Materials & experimental systems

n/a	Involved in the study	
☐	☒	Antibodies
☐	☒	Eukaryotic cell lines
☒	☐	Palaeontology and archaeology
☐	☒	Animals and other organisms
☒	☐	Clinical data
☒	☐	Dual use research of concern
☒	☐	Plants

Methods

n/a	Involved in the study	
☒	☐	ChIP-seq
☒	☐	Flow cytometry
☒	☐	MRI-based neuroimaging

Antibodies

Antibodies used	CR3022, S309, H014, ADG-2, CC40.8, B6, S2X259

Validation	Genes encoding the above antibodies were synthesised at Genscript and were expressed and puried from Expi293f cells. Antibodies were used for SPR and ELISA experiments.

Eukaryotic cell lines

Policy information about cell lines and Sex and Gender in Research

Cell line source(s)	Expi293F cell line: Thermo fisher scientific Gibco™ A14528
Authentication	Purchased from Thermo fisher scientific Gibco™ A14528
Mycoplasma contamination	Cell line tested negative for mycoplasma contamination.
Commonly misidentified lines (See ICLAC register)	No misidentified cell line was used.

Animals and other research organisms

Policy information about studies involving animals; ARRIVE guidelines recommended for reporting animal research, and Sex and Gender in Research

Laboratory animals	6-8 weeks old, female BALB/c mice, hACE-2 expressing C57BL/6 transgenic mice, golden Syrian hamsters
Wild animals	The study did not involve wild animals
Reporting on sex	Female mice and hamsters were used in the study.
Field-collected samples	This study did not involve samples collected from the field.
Ethics oversight	All mice and Hamster immunization studies were approved by the Institutional Animal Ethics Committee (Approval numbers CAF/ETHICS/847/2021; CAF/ETHICS/887/2022). These were carried out at the Central Animal Facility (CAF), Indian Institute of Science, according to CPCSEA and ARRIVE guidelines.

Note that full information on the approval of the study protocol must also be provided in the manuscript.
